# Dual-Band Wearable MIMO Antenna for WiFi Sensing Applications

**DOI:** 10.3390/s22239257

**Published:** 2022-11-28

**Authors:** Sima Noghanian

**Affiliations:** CommScope Ruckus Networks, Sunnyvale, CA 94089, USA; sima_noghanian@ieee.org

**Keywords:** WiFi antenna, textile antenna, MIMO antenna

## Abstract

Multiple input multiple output (MIMO) technology combined with orthogonal frequency division multiple access (OFDMA) is an enabling technology used in WiFi 6/6E (IEEE 802.11ax) to increase the throughput. With the addition of WiFi 6/6E and taking advantage of MIMO and OFDMA, many applications of wearable WiFi can be imagined. For example, WiFi can be used for tracking and fall detection. Wearable devices, such as those used in gaming, vital sign monitoring, and tracking, can also take advantage of wearable MIMO antennas. In this paper, a wearable small dual-band antenna is proposed that can be fabricated on felt or denim substrate. In the proposed antenna, a conductive layer is used as a reflector to improve the gain and reduce the sensitivity of the antenna performance to the body loading effects. The details of the design and its performance in a sample indoor MIMO setting are provided. The MIMO antenna is proposed for WiFi tracking and sensing applications. The performance of the MIMO antenna in an indoor setting is examined.

## 1. Introduction

Wireless connectivity plays a major part in our daily lives. The wireless local area network (WLAN) is one of the major technologies to provide this connectivity. This is even more evident now than before, due to the increase of sensors and Internet of Things (IoT) devices that are controlled over the WLAN. Most WLANs operate under IEEE 802.11 standards, referred to as WiFi networks. With the focus on wearable wireless devices, it is desired to have a direct connection to networks such as WiFi. Therefore, there is a need for wearable antennas in the WiFi bands that can seamlessly be integrated into clothing. Such antennas should be small. However, the small antennas usually have omnidirectional patterns, and their input impedance may be affected significantly by the lossy human tissue in their vicinities. On the other hand, the radiation toward the body can cause a high specific absorption rate (SAR), requiring a reduction in the input power to comply with the standard SAR levels. 

The goal of this work was to provide a small antenna design with good isolation from the wearer’s body tissue. The target application is WiFi sensing. WiFi sensing is used in three major applications: (1) detection (e.g., motion, fall, and sleep detection), (2) recognition (e.g., activity recognition, exercise), and (3) estimation (e.g., position, orientation, tracking, gait). In many of these applications, multiple antennas are required or provide a more accurate result. Therefore, we are looking for a multiple input multiple output (MIMO) system comprised of largely separated textile antennas, distributed on the body.

There are three categories of textile antennas:

- Planar monopole or patch antennas: Small size and wide bandwidth are the advantages of this category, but the low gain, sensitivity to the loading effects of the human body, and the omnidirectional patterns are the disadvantages of it [1,2].
- Planar monopole on a periodic structure: Using the periodic structure that acts as an artificial magnetic conductor (AMC), the radiation toward the body is reduced. Therefore, the SAR level is reduced, and the gain is increased. However, AMC requires a large area, and the choices of locations of the antenna become limited to the larger parts of the body [3,4,5,6,7,8,9].
- Semi-rigid or rigid antennas in the form of buttons, a part of a wristband, belt, or eyewear: The use of a rigid substrate with a higher dielectric constant helps to reduce the size and improve the rigidity of the design. However, there are specific and limited locations where this antenna type can be used [10,11].

The frequency bands of interest follow the 802.11 a/b/g/n/ac/ax 2.401–2.484 GHz (referred to as 2.4 GHz herein) and 5.170–5.835 GHz (referred to as 5.5 GHz herein). Table 1 summarizes some of the wearable designs and compares their volume, gain, and efficiency. The goal of this research was to find a small-size textile antenna that can be located on different parts of the body to form a MIMO system that can communicate with WiFi access points (APs) with different polarizations. A wide beam broadside pattern was desired. Considering the three categories of antennas that were discussed, planar monopole antennas, although they are small, are not desirable for this purpose. The antenna can easily be affected by the body tissue properties and movement of the person. The resonance frequency may change, in which case the antenna becomes inefficient. The periodic structures are also not used due to the requirement of large size. The idea in this work is to have a small antenna that can easily be integrated into clothing and spread around the body to provide rich polarization diversity. Finally, the semi-rigid structure was not considered due to the limitation on the locations in the clothing. In summary, the proposed antenna has the following advantages: (1) It is made of textile and does not require any rigid dielectric, (2) it is compact while providing high gain, and (3) it has low back radiation without using a large periodic structure.

The rest of the paper is organized as follows: After the introduction, the structure of the antenna is explained in detail. The effects of the substrate and air gap between the antenna and the body on the antenna matching are presented. The effects of bending, the gain, and SAR in different cases are reported and the measurement results of the fabricated antenna are discussed. The final section introduces the WiFi sensing application and studies capacity improvement using a MIMO wearable antenna system in a small indoor environment.

## 2. Antenna Design and Performance 

### 2.1. Antenna Design

**Figure 1:**
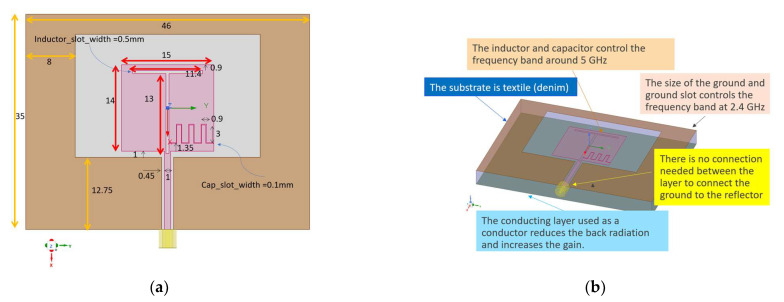
Proposed dual band antenna structure: (**a**) Top view of the antenna, all dimensions are given in mm; (**b**) Major features of the design.

A textile antenna that operates in the two mentioned WiFi frequency bands is desired. The proposed design in this paper is a combination of a patch and a ring around it. Figure 1 shows the antenna structure. The patch incorporates a narrow T shape slot that creates an inductor and an inter-digital capacitor. The design is inspired by [14], but it has major differences. Since the antenna is designed to be on a flexible substrate, the dependency on the substrate thickness and loading effects of the lossy human tissues should be considered. First, the antenna was designed on a denim substrate with the relative permittivity of $\varepsilon_{r}=1.5$ and loss tangent of tan δ=0.0006. The thickness of the denim is considered to be h, which needs to be optimized for the frequency bands of WiFi. To consider the effects of the human body in the simulation a block of human average tissue material with the permittivity of $\varepsilon_{r}=35$ and conductivity of σ=4 S/m was considered. To consider the effect of the air and clothing between the antenna substrate and the body surface, in the simulation an air gap between the antenna and the tissue block was considered. Since the clothing usually has low relative permittivity or dielectric constant, it can be modeled as an air layer. Therefore, the combination of the air and textile is considered as the air gap. The second major difference between the proposed design and the antenna in [14] is the addition of a conducting reflector in the back of the substrate, to isolate the antenna and stabilize the resonance frequency. The third major difference is the mechanism of radiation at the low-frequency band. The ground was extended as a ring around the main patch. This ring was added to improve the low-frequency operation and stabilize the resonance frequency at the 2.45 GHz band.

**Figure 2:**
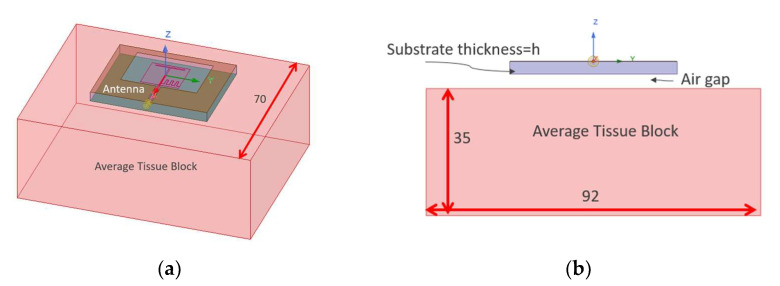
The antenna was placed on a block of average tissue: (**a**) Tissue block and antenna placement; (**b**) Side view, all dimensions are in mm.

The reflection coefficient (S11) variation due to substrate thickness “h” and “air−gap” changes were studied. Figure 2 shows the simulation model. The antenna is placed on a block of human average tissue. The tissue block has the dimensions of $92\times 70\times 35{\;\mathrm{mm}}^{3}$.

### 2.2. Substrate and Air Gap Effects

**Figure 3:**
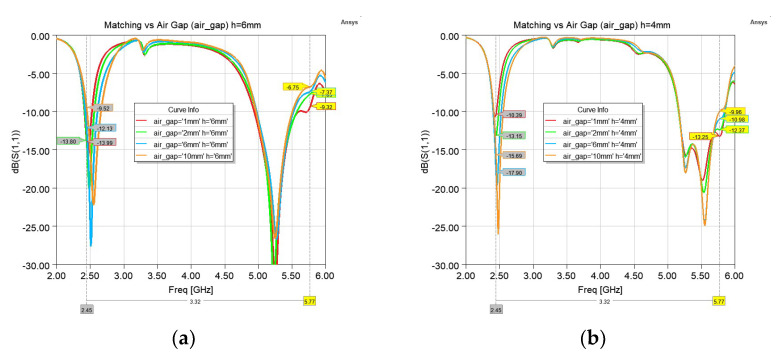
Reflection coefficient (S11) variation with air−gap size for (**a**) Substrate thickness of 6 mm; (**b**) Substrate thickness of 4 mm.

By the addition of the reflector plane, the antenna will be mostly isolated from the tissue layer and matching will not be affected by the variation of the air−gap. This is shown by simulating the antenna on the tissue phantom by varying the air−gap for two substrate thicknesses of 4 mm and 6 mm. All simulations in this paper were performed using ANSYS Electronics Desktop 2021.R2. As shown in Figure 3, a minimum of air−gap of 2 mm provides an acceptable bandwidth. In general, the lower band (referred to as the 2.4 GHz band), as well as the lower side of the higher band (referred to as the 5.5 GHz band), are less affected by the air-gap values. Most of the sensitivity is for frequencies above 5.5 GHz.

**Figure 4:**
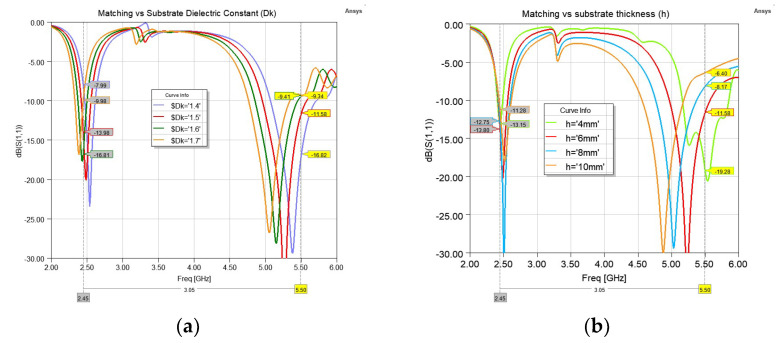
Reflection coefficient variation for various (**a**) Substrate dielectric const (Dk); (**b**) Substrate thicknesses (h) while air−gap was kept at 2 mm.

**Figure 5:**
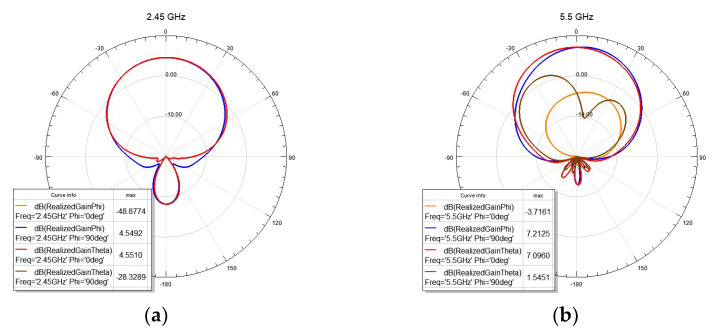
Radiation patterns for the antenna on a 6 mm substrate with a 2 mm air−gap in the flat condition: (**a**) 2.45 GHz; (**b**) 5.5 GHz.

As expected, the antenna shows more sensitivity to the variations in the substrate size and material. The dielectric constant of the substrate might change due to humidity. To show the effect of the variation in the dielectric constant, it was changed from 1.4 to 1.7 and the reflection coefficient is shown in Figure 4a. As expected, a shift in the resonance frequency is observed. The change of dielectric thickness also has severe effects, as it is causing the height from the reflector to be changed. To show this effect, Figure 4b shows the reflection coefficient when the air−gap is kept at 2 mm, but the substrate thickness (h) is changed from 4 mm to 10 mm. The 2.4 GHz band is less sensitive to this change, but the 5.5 GHz band can be affected. The optimum thickness is around 5 mm, but for a textile antenna, it is not always possible to keep the thickness at a constant value due to stretching and pressure. For the rest of the study, a thickness of 4–6 mm is considered. Figure 5 shows the radiation patterns in the two E- and H-planes.

### 2.3. Specific Absorption Rate and Gain

**Figure 6:**
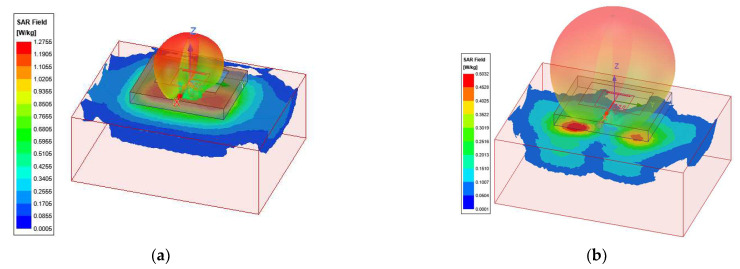
SAR values for the proposed antenna on a block of average tissue, using 1-g average SAR and input power for 50 mW: (**a**) 2.45 GHz; (**b**) 5.5 GHz.

Since this antenna is considered for wearable WiFi applications, it is important to study the specific absorption rate (SAR) for different conditions. For 6 mm substrate thickness and air−gap=2 mm, the gain and maximum specific absorption rate (SAR in W/kg) for a 50 mW input power were calculated and summarized in Table 2. Table 2 also summarizes the antenna efficiency and bandwidths. Similarly, these values for the antenna with 4 mm substrate thickness are given in Table 3. The SAR distribution for the case of h=6 mm and at 2.45 GHz and 5.5 GHz are shown in Figure 6.

### 2.4. Bending Effects

**Figure 7:**
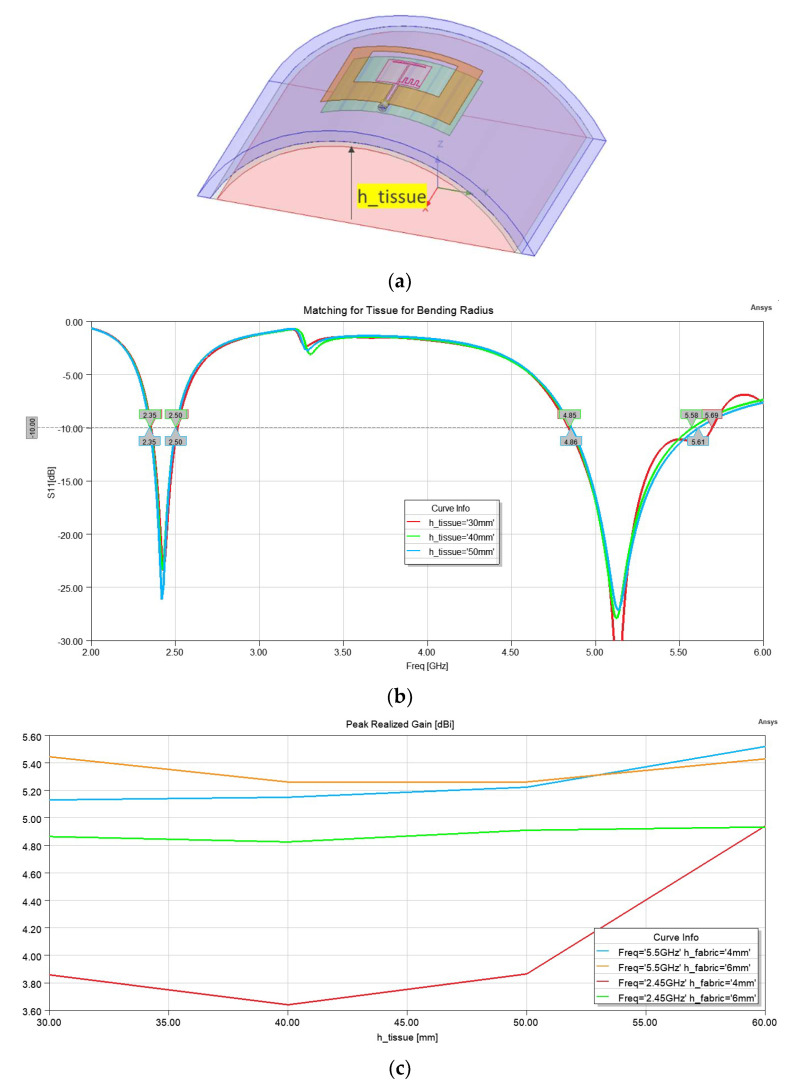
Antenna performance under bending condition (simulation): (**a**) Bending profile; (**b**) S11 under various bending radii (h_tissue); (**c**) gain variation vs. bending radii (h_tissue) for the antenna on 4 mm and 6 mm substrates at 2.45 GHz and 5.5 GHz.

One of the major causes of the degradation of textile antenna performance is the bending effects. Depending on the location of the antenna on the wearer’s body, it may be bent. We considered the effect of bending when it was bent along the H-plane. To simulate this effect, a cylindrical block of tissue with a radius of “h_tissue” was considered. The air-gap was added by adding a 2 mm cylindrical shell of air, and then the reflector layer and substrate, and antenna conductive layers were added. Figure 7a shows the simulation setup. Figure 7b shows the effect of bending on the reflection coefficient for a 2 mm air−gap and 6 mm substrate thickness. Finally Figure 7c shows the effect of the bending on the gain, at 2.45 GHz and 5.5 GHz, for a substrate thickness of 4 mm and 6 mm, as h_tissue was changed from 30 mm to 60 mm. This figure shows that the 2.45 GHz band is more affected by the bending than the 5.5 GHz.

**Figure 8:**
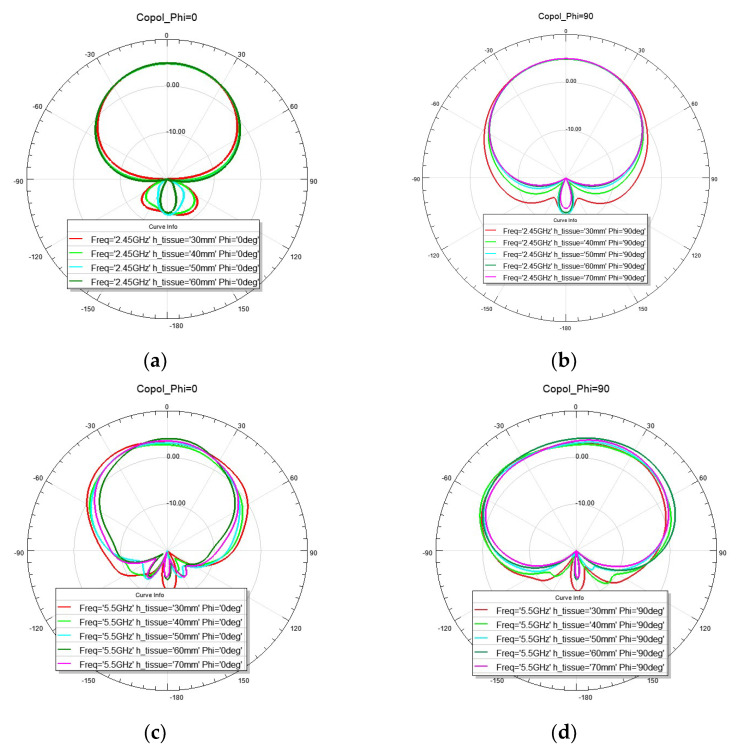
Simulated co-polarization radiation patterns for bending condition: (**a**) frequency 2.45 GHz *phi* = 0 degrees; (**b**) frequency 2.45 GHz phi=90 degrees; (**c**) frequency 5.5 GHz phi=0 degrees; (**d**) frequency 5.5 GHz phi=90 degrees.

Radiation patterns on the E- and H-planes of the antenna under bending are shown in Figure 8. It can be seen that the pattern on the H-plane which is the plane of bending starts to widen. Moreover, one may notice that as the radius of bending becomes smaller, there is more back radiation and the gain is slightly decreased.

## 3. Measurement Results

### 3.1. Antenna on Phantom

**Figure 9:**
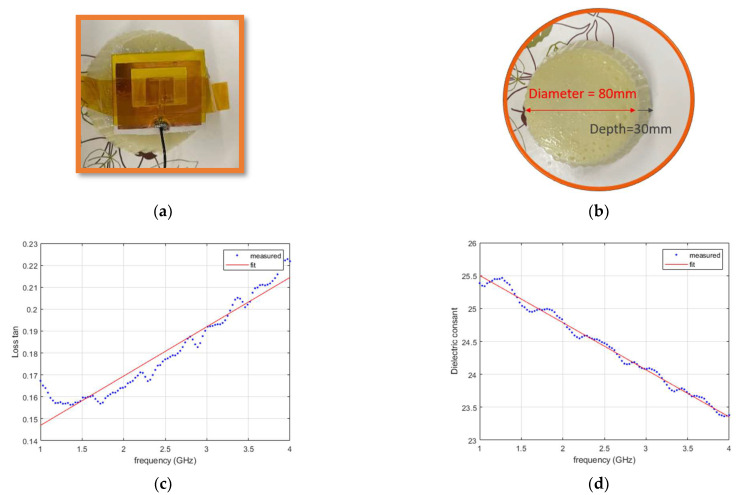
Tissue phantom: (**a**) fabricated antenna on the phantom; (**b**) phantom dimensions; (**c**) loss tangent and, (**d**) dielectric constant measurement of the fabricated antenna using the dielectric probe.

The antenna was fabricated using substrate materials of foam and conductor layers of copper tape. The patch and the slots were cut out of the copper tape using Silhouette Cameo 4 cutting machine. Figure 9a shows the fabricated antenna that is placed on the phantom layer. A tissue phantom was made using 70 mL of distilled water, 70 mL of oil, 7 g of gelatin powder, and 4 mL of dish soap. First, the gelatin powder was dissolved in the distilled water by slowly heating the mixture to about 85 °C. Then, the heat was turned off and the mixture left to cool down to 65 °C. Oil was also heated to 65 °C. Then, the gelatin mixture and oil and liquid dish soap were mixed until they formed a uniform liquid. The stirring of the mixture continued until the temperature dropped to 50 °C. Then, the mixture was transferred to a covered container and left at room temperature overnight. The resulting phantom is shown in Figure 9b. The phantom was a cylindrical block with a diameter of 80 mm and a depth of 30 mm. The phantom material properties were measured using Keysight Dielectric Performance Probe (at NeuSpera Medical). The measured and fitted curves for the dielectric loss tangent are shown in Figure 9c and the dielectric constant is shown in Figure 9d. The fitted curve that was used in the simulations is given In Table 4. The substrate material measurement was also performed. Although for low loss material, such as foam and felt fabric, the probe does not give an accurate measurement, results show the assumed values in the simulations are not too far from the measured values. Table 4 summarizes the results of the curve fitting for each material.

**Figure 10:**
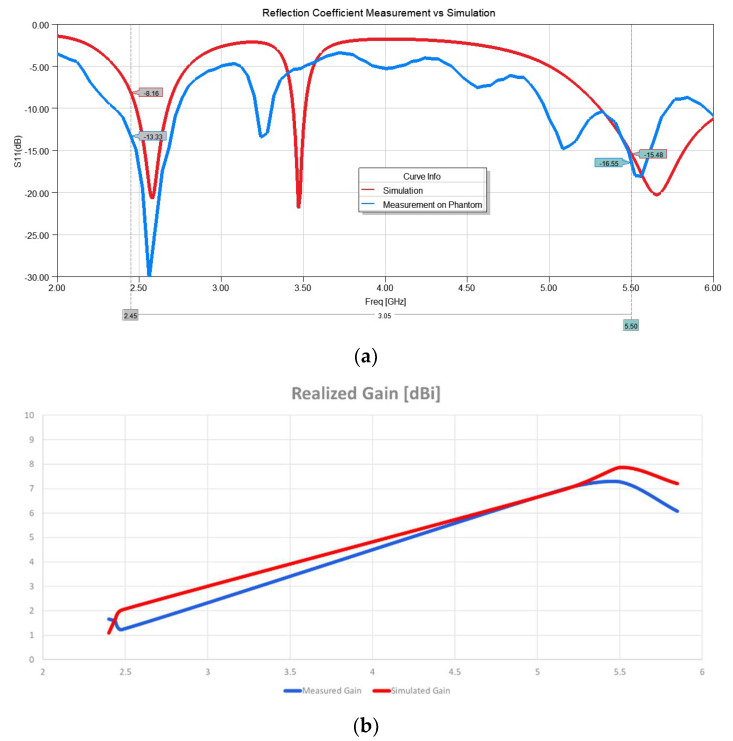
Antenna measurement on phantom: (**a**) S11 measured vs. simulated; (**b**) gain measured vs. simulated.

After characterizing the material, the simulation was modified to include the fitted curve of the dielectric constant and loss tangent for the phantom. The simulation showed that the length of the antenna had to be increased from 35 mm to 37 mm since the substrate material was slightly different than the original design. After making the adjustment and running the simulation, the measured reflection coefficient and the simulated ones were compared, as shown in Figure 10a. A Copper Mountain Vector Network Analyzer was used for this measurement. The gain was measured using a StarLab antenna measurement system. The simulated and measured gain results are shown in Figure 10b. A good agreement between simulation and measurement is observed.

### 3.2. Antenna on Human Body

**Figure 11:**
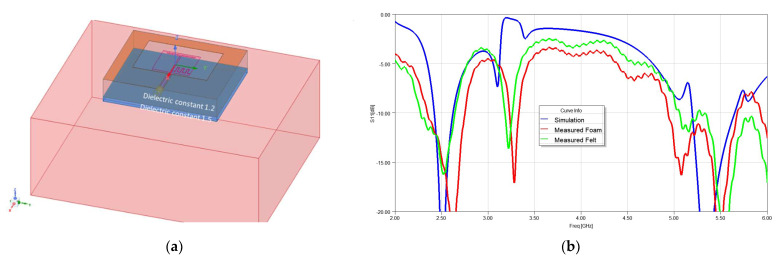
Antenna measurement on body considering the layer of cloth (**a**) assumption of the layers in the simulation; (**b**) measured S11 for the antenna on foam and felt substrates vs. simulation.

The antenna was then placed on a human foot wearing denim pants. The denim thickness was assumed to be approximately 1.5 mm. Two cases of foam and felt fabric used as the substrate were considered and S11 was measured. Figure 11a shows the simulation set up and Figure 11b shows the comparison between the measured and simulated S11.

## 4. Wearable WiFi MIMO Performance

### 4.1. WiFi Sensing and Wearable Antennas

**Figure 12:**
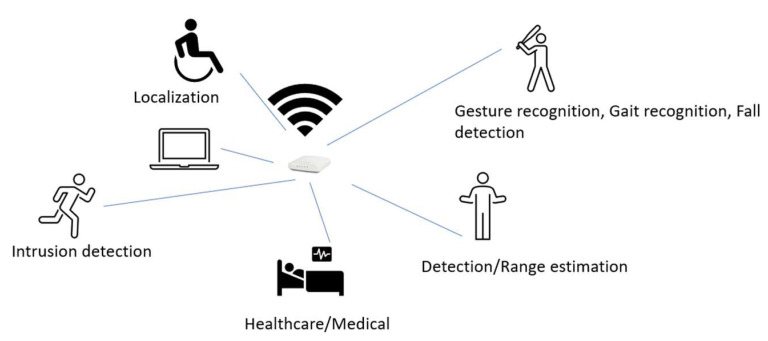
WiFi sensing may be used for many different applications: Localization, intrusion detection, health and medical monitoring, range detection and estimation, gesture recognition, gain recognition, and fall detection.

One of the applications of wearable antennas such as the one proposed here is WiFi sensing. WiFi sensing is a relatively new technology that provides added capability to WiFi used for applications such as motion detection, gesture recognition, biomedical vital signal monitoring, fall detection, and many other applications (Figure 12). WiFi sensing is based on two basic methods: the passive method, and the triggered method. In the passive method, a radar approach is taken. The WiFi access point (AP, or receiver device) sends a data frame and estimates the location of the station (STA, or illuminator device) from the acknowledge frame that is sent back. This method although does not require additional hardware or software on the STA side is less accurate and requires additional radar signal processing on the AP side. The STA does not have any control over the rate of transmission and its characteristics and cannot turn the sensing off. In the second approach, the WiFi sensing device is triggered to transmit a WiFi packet to be used for WiFi sensing. This method is more accurate and provides more control. Two APs may be used as the receiver and illuminator devices.

**Figure 13:**
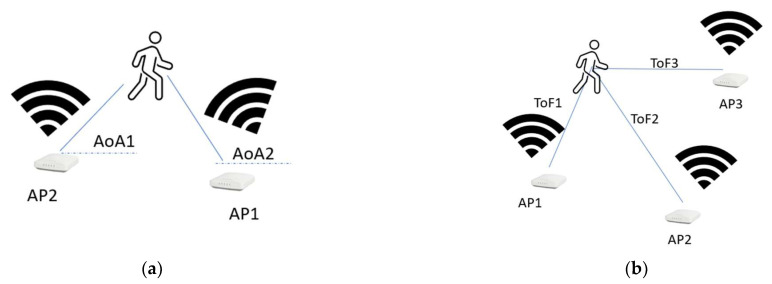
WiFi sensing with different situations; (**a**) using two APs and measuring Angle of Arrival (AoA); (**b**) Using 3 APs and measuring Time of Fly (ToF).

The sensing may be conducted based on the received signal strength indicator (RSSI) [15], or by using more detailed channel information obtained from Channel State Information (CSI) [16]. In CSI, the channel response, angle of arrival, and time of fly are extracted based on the multi-path information (Figure 13). Therefore, having more diverse antennas can provide more precise channel information. However, in most cases, the MIMO antennas are closely located on one device and have very small space diversity.

**Figure 14:**
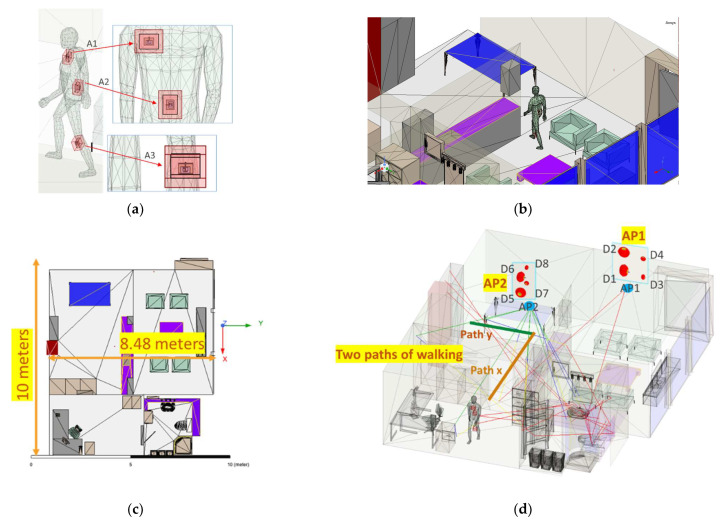
Shooting-bouncing ray (SBR+) simulation set up: (**a**) three textile antennas were placed on a human body of average tissue material; (**b**) a human model was placed inside the indoor office area and moved around; (**c**) office area dimensions; (**d**) location of the access points (AP) and the paths of moving the body.

Using wearable antennas that are located on different parts of the body of the wearer provides space diversity as well as pattern and polarization diversity. There is no need for the antennas to be attached to a phone or laptop. They could be integrated into a vest, hat, or other wearables. These small antennas will form a MIMO antenna system that is used for WiFi sensing (Figure 14a). The antennas will communicate with the APs directly. In WiFi-triggered sensing, the AP is talking to different STAs, such as phones and computers in the room. Therefore, there is an issue of privacy and dependency on the locations of STAs. The use of wearable antennas will remove this condition.

### 4.2. Performance of Wearable MIMO Antenna

The performance of the proposed dual-band wearable antenna is examined in different MIMO settings considering a shooting-bouncing-rays (SBR+) analysis using ANSYS Electronics Desktop (AEDT 2021R2). A small indoor office with two AP locations is used for this study (Figure 14b). The total area of the office is 10 m ×8.48 m, and various objects, such as doors, walls, chairs, and tables, are in the office (Figure 14c). The objects in the room are made of a material such as glass, wood, and metal. One AP is located on the wall at a height of 1.5 m. The second AP is on the ceiling at a height of 2.7 m. Each AP has 4 antennas, to provide 2 polarizations and 2 frequencies. The spacing between the antennas is 5 cm. AP1 has two dipole antennas at 2.45 GHz (D1 and D2) and two dipoles at 5.50 GHz (D3 and D4). The antennas on the ceiling are named D5 and D6 for the 2.45 GHz dipoles and D7 and D8 for the 5.50 GHz dipoles (Figure 14d).

A human body that was filled with the same average tissue properties (the permittivity of $\varepsilon_{r}=35$ and conductivity of σ=4 S/m) as it was considered during the antenna design is in this room wearing 3 wearable antennas. The antenna locations on the human subject are: (A1) on the chest at the height of 1.4 m, (A2) on the back at the height of 1.1 m, and (A3) on the lower part of the leg. A1 and A2 have 90° orientation differences. A3 is at an angle of 110° and has the same orientation as A1. The body was moved along path x and path y, shown in Figure 14d. For each location different combinations of the antennas were considered to create a 2×2 MIMO setting. It is worth mentioning that in these simulations, antennas are not covered by any protective layers and the effects of humidity, dust, and other environmental effects are ignored.

First, the channel matrix (H) was created using the Sji values given by the SBR+ simulations. Assuming the total transmit power is equally allocated to all *n* antennas, the capacity of n×n MIMO is given by [17]:

(1) $$C={log}_{2}det\left[I_{n}+\frac{\rho }{n}HH^{H}\right]$$

where $H^{H}$ is the conjugate transpose of H, and the average received signal-to-noise ratio is defined as $\frac{\rho }{n}$.

**Figure 15:**
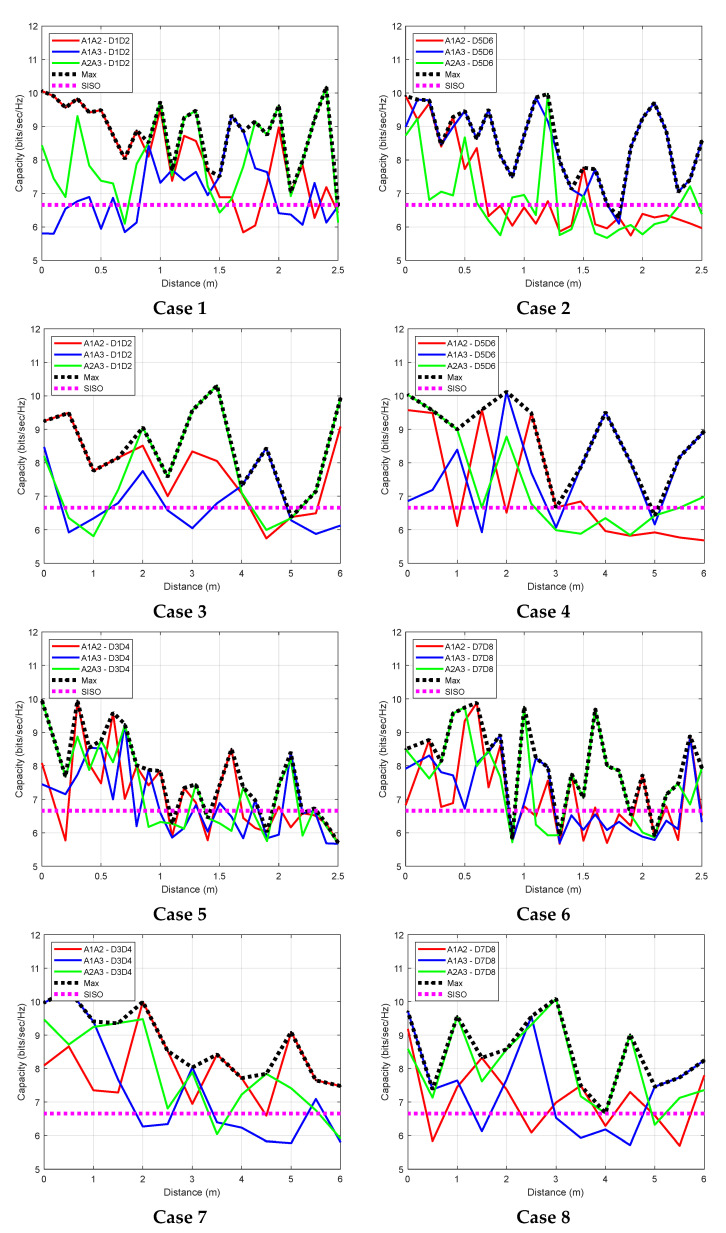
Capacity calculated for various MIMO settings.

Figure 15 shows the capacity (in bits/s/Hz) for various locations for any combination of 2×2 MIMO antenna system, as the person moves along the x and y paths. For any given position, the maximum capacity is also shown. There are eight total cases, depending on the frequency and the path taken. Table 5 gives a summary of the capacity for each case, as well as the maximum capacity, if the system can pick the best combination. In this case, the percentage of the increase in capacity over single input single output (SISO) is from 16% to 33%. This case study shows how by distributing the antennas on the body, the capacity can improve. However, there are many other ways to use the distributed antennas, e.g., by computing the angle of arrival and time of fly, an accurate location can be found. This can be used for applications such as fall detection.

## 5. Discussion

The use of a textile antenna provides access to a large area of the body to create different MIMO sets. Due to the possibility of increasing the distance, the space-diversity can be achieved much easier than compact MIMO antennas in handheld or small portable devices. The other possibility is the use of different orientations to create polarization diversity. The combination of space and polarization diversity can create orthogonality in the wireless channels and improve capacity.

Enabling WiFi sensing in personal devices can be subject to privacy concerns and there may be hesitance in including it in a personal device, such as a cellphone or tablet. The other advantage of using a textile antenna with a stand-alone device is that the WiFi sensing can be integrated into a small vest or jacket and the person can choose to wear it or not. This will assuage privacy concerns.

Both areas of WiFi sensing and wearable antennas are relatively new areas of technology development. More research needed to understand the best configuration of the antenna placement and methods of selecting the best MIMO antenna elements. The surface waves that travel through the body tissue can create coupling between the antennas [18]. This may affect the antenna location and orientation selection.

The future direction of this research is to study the performance of MIMO antennas with various antenna selection methods, considering the effects of surface waves and environmental factors.

## 6. Conclusions

A dual-band WiFi wearable antenna is proposed and studied. The antenna provides a small form factor with a high gain and small back radiation to reduce SAR values. The antenna is designed on a denim substrate and occupies only a 35 mm by 46 mm area. It covers both 2.45 GHz and 5.5 GHz WiFi bands. It can provide 4.5 dBi gain at 2.45 GHz and 7.4 dBi at 5.5 GHz, when the substrate thickness is 6 mm and space is 2 mm from the body. The SAR value for 50 mW input power for 2.45 GHz (5.5 GHz) for 1 g-average SAR is 1.275 (0.503) W/kg. As for the 10-g-average tissue SAR, the maximum value is 0.585 (0.154) W/kg at 2.45 GHz (5.5 GHz).

A study of the use of an antenna on a human body model with three possible locations to form a selection of 2×2 MIMO set up for a small office area was performed through a shooting-bouncing ray simulation. The simulation showed that, along the two selected paths, the increase in capacity can be up to 33%.

## 7. Patents

The following provisional patents result from the work reported in this manuscript:

- WEARABLE ANTENNAS FOR WIFI COMMUNICATIONS, US Application No. 63/381,179, filed on 27 October 2022 in the United States Patent and Trademark Office via the Electronic Filing System.
- WEARABLE ANTENNAS FOR WEARABLE WIFI SENSING SYSTEM, US Application No. 63/381,177, filed on 27 October 2022, in the United States Patent and Trademark Office via the Electronic Filing System.

## Figures and Tables

**Table 1:** Comparing the WiFi wearable antennas proposed in the literature and this paper.

Ref.	Antenna Type	Substrate Material (Dielectric Constant, Loss Tangent)	Size [mm^3^]	Gain [dBi] (@2.45 or Lower Band, @5.5 or Upper Band)/	Efficiency % (@2.45 or Lower Band, @5.5 or Upper Band)/
[1]	Monopole	Felt (1.3, 0.044)	37.20 × 50.00 × 1.50	3.03, 4.8	83, 51 (in air) 53, 51 (on body)
[2]	CPW fed Monopole	Felt (1.22, 0.016)	60.00 × 30.00 × 2.00	1.3, 5.0	?
[5]	Microstrip on AMC Antenna 1	Polyethylene Foam (1.05, ?)	176.50 × 176.50 × 6.00	8.4, 11.4	97, 96 (in air)
[5]	Microstrip on AMC Antenna 2	Polyethylene Foam (1.05, ?)	136 × 136.00 × 6.00	9.4, 9.1	95, 94 (in air)
[6]	Monopole on AMC	Phelan (1.08, 0.008)	102.00 × 68.00 × 3.60	-, 6.1	?
[7]	Monpole	Felt (1.22, 0.016)	85.00 × 85.00 × 2.00	-	?
[8]	CPW fed Monopole on AMC	RO4003 (3.38, 0.0027)	43.20 × 43.20 × 4.60	NA, 8.2	?
[9]	CPW fed Monopole on AMC	Textile (1.38, 0.02)	120.00 × 120.00 × 4.48	6.4, 7.6	?
[10]	Button Planar Inverted F	Combination RO5880 (2.2, 0.0009), Felt (1.4, 0.044)	45.00 × 45.00 × 6.83	2.1,6.7	72, 92 (in air) 65, 82 (on body)
[11]	Button Planar Inverted F	Combination RO5880 (2.2, 0.0009), Textile (1.4, 0.044)	80.00 × 80.00 × 5.97 (Button radius 7.26 mm)	1.1, 6.0	~90 (in air)
[12]	Quasi-Yagi	RO4350b (3.48, 0.0039)	55.00 × 48.00 × 0.25	6.8, 5.3	?
[13]	MagnetoElectric Dipole	Felt (1.3, 0.044)	100.00 × 100.00 × 6.00	3.0, 4.7	50, 60 (in air)
Proposed	MIMO, CPW Monopole on Reflector Surface	Denim (1.5, 0.0006)	35.00 × 46.00 × 6.00	4.5, 7.4	40, 60 (on body)

**Table 2:** SAR and antenna gain and bandwidth summary for h = 6 mm and air−gap = 2 mm.

Frequency (GHz)	Realized Gain (dBi)	Radiation Efficiency %	1-g Avg. SAR (W/Kg)	10-g Avg. SAR (W/Kg)	Bandwidth (MHz)
2.45	4.55	50.19	1.275	0.585	140
5.50	7.39	86.46	0.503	0.154	610

**Table 3:** SAR and antenna gain and bandwidth summary for h = 4 mm substrate and air−gap=2 mm.

Frequency (GHz)	Realized Gain (dBi)	Radiation Efficiency %	1-g Avg. SAR (W/Kg)	10-g Avg. SAR (W/Kg)	Bandwidth (MHz)
2.45	3.80	43.31	1.465	0.670	80
5.50	8.40	86.76	0.581	0.193	690

**Table 4:** Phantom and substrate material properties curves fitted to the measured values; “*f*” is the frequency in GHz.

Material	Relative Permittivity	Loss Tan
Phantom	26.22000−0.7169f	0.12450+0.02249f
Substrate	1.16300−0.01040f	0.04444−0.00223f

**Table 5:** Summary of the capacity of 2 × 2 MIMO combination when the person moves along the x or y directions, all capacity values are in bits/s/Hz.

Case #	AP Antennas	Freq (GHz)	Path	Wearable Antennas			Max ^1^	% Increase over SISO
				A1A2	A1A3	A2A3		
1	D1D2	2.45	y	8.10	7.00	8.05	8.87	33
2	D5D6			7.00	8.41	6.79	8.53	28
3	D1D2		x	7.79	6.82	7.73	8.49	27
4	D5D6			7.19	7.76	7.30	8.72	31
5	D3D4	5.50	y	7.11	6.88	7.17	7.70	16
6	D7D8			7.08	7.02	7.58	8.07	21
7	D3D4		x	7.99	7.32	7.86	8.76	16
8	D7D8			7.11	7.38	8.04	8.45	16

^1^ MIMO combination with the maximum capacity was chosen.
